# Morphological, physiological and anatomical traits of plant functional types in temperate grasslands along a large-scale aridity gradient in northeastern China

**DOI:** 10.1038/srep40900

**Published:** 2017-01-20

**Authors:** Chengyuan Guo, Linna Ma, Shan Yuan, Renzhong Wang

**Affiliations:** 1 State Key Laboratory of Vegetation and Environmental Change, Institute of Botany, the Chinese Academy of Sciences, 20 Nanxincun, Xiangshan, Beijing, 100093, China; 2 University of Chinese Academy of Sciences, 19 Yuquan Road, Beijing, 100049, China

## Abstract

At the species level, plants can respond to climate changes by changing their leaf traits; however, there is scant information regarding the responses of morphological, physiological and anatomical traits of plant functional types (PFTs) to aridity. Herein, the leaf traits of five PFTs representing 17 plant species in temperate grasslands were examined along a large-scale aridity gradient in northeastern China. The results show that leaf thickness in shrubs, perennial grasses and forbs increased with heightened aridity. Trees increased soluble sugar content, but shrubs, perennials and annual grasses enhanced proline accumulation due to increasing aridity. Moreover, vessel diameter and stomatal index in shrubs and perennial grasses decreased with increasing aridity, but stomatal density and vascular diameter of five PFTs were not correlated with water availability. In conclusion, divergences in adaptive strategies to aridity among these PFTs in temperate grasslands were likely caused by differences in their utilization of water resources, which have different temporal and spatial distribution patterns. Leaf traits of shrubs and perennial grasses had the largest responses to variability of aridity through regulation of morphological, physiological and anatomical traits, which was followed by perennial forbs. Trees and annual grasses endured aridity only by adjusting leaf physiological processes.

At global and regional scales, the increase in aridity poses the most important environmental threat to plant survival, productivity and vegetation dynamics^1^,^2^,^3^,^4^. Over the last few decades, many studies have been published to investigate the effects of aridity on plant species, including plant biomass allocation^5^, morphological traits^6^,^7^, physiological processes and anatomical structures^8^,^9^ in response to water availability gradients. Plants employ three common strategies to adapt to water scarcity; escape, tolerance and avoidance^1^. Escape involves successful reproduction before the onset of severe stress, while avoidance relies on delayed initiation of water scarcity in plant tissues, and tolerance is a result of coordinated physiological and biochemical alterations at the cellular and molecular levels^1^,^8^. These strategies are not mutually exclusive, and plants can even use combined strategies^10^. In the long-term, plants develop these strategies through a variety of adaptive traits involving minimization of water loss and maximization of water uptake. Water loss is minimized by changing stomatal traits^11^, increasing leaf mass per area (LMA), thickening leaf blades^12^, and enhancing proline and soluble sugar contents in leaf cells to maintain water balance and cell turgor under water scarcity^13^,^14^,^15^,^16^. Meanwhile, water uptake is maximized by increasing investment in vessel number and size in stems and enlarging vessel diameter in leaves^17^,^18^. Most previous studies related to these strategies were based on individual species; however, there is little literature examining the strategy divergences among plant functional types (PFTs) along large-scale aridity gradients.

PFTs are defined as groups of plants exhibiting either similar responses to an environment or similar effects on major ecosystem processes based on plant morphology, physiology and life history^19^,^20^. Grouping large numbers of species that are functionally similar into a single classification greatly reduces the complexity of the intrinsically hierarchical nature of ecosystems^21^ and bridges the gap between plant physiology and ecosystem processes^22^. Therefore, PFTs might be more useful for ecosystem responses to climate changes at large scales than plant species. A better understanding of PFTs responses to variation in aridity is crucial for regions with water scarcity, such as farming pastoral zones, grasslands and deserts, and is important to the development of better land management practices to adapt to such changes.

Northeast China grasslands, which cover an area of about 8.6 million ha, are located at the eastern end of the Eurasian steppe zone. This region is characterized by a natural aridity gradient that provides an ideal system to conduct such a study. PFT composition^23^, classification^24^ and diversity^25^ in the region have been well documented, but leaf anatomy and physiology have not been studied. In this study, we selected 17 species present in all sites along a large-scale aridity gradient. We grouped these into five PFTs on the basis of life form and plant size^23^,^24^ and measured the phenotypic plasticity within each species. We hypothesize that water availability is the critical factor driving trait variations of PFTs and that there are significant divergences in morphological, physiological and anatomical traits among PFTs to cope with water scarcity along the large-scale gradient.

## Results

**Figure 1:**
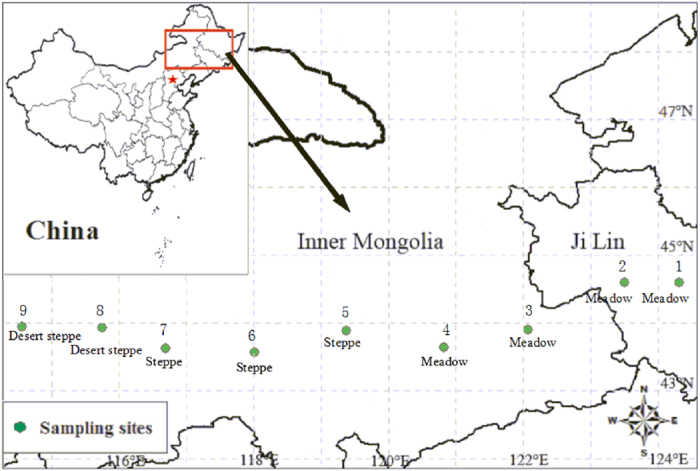
Sample locations (see Table 1) in temperate grasslands along a large-scale aridity gradient in northeast of China. The map was generated by ArcGIS.9.3.SLX (http://www.esri.com/software/arcgis/).

**Figure 2:**
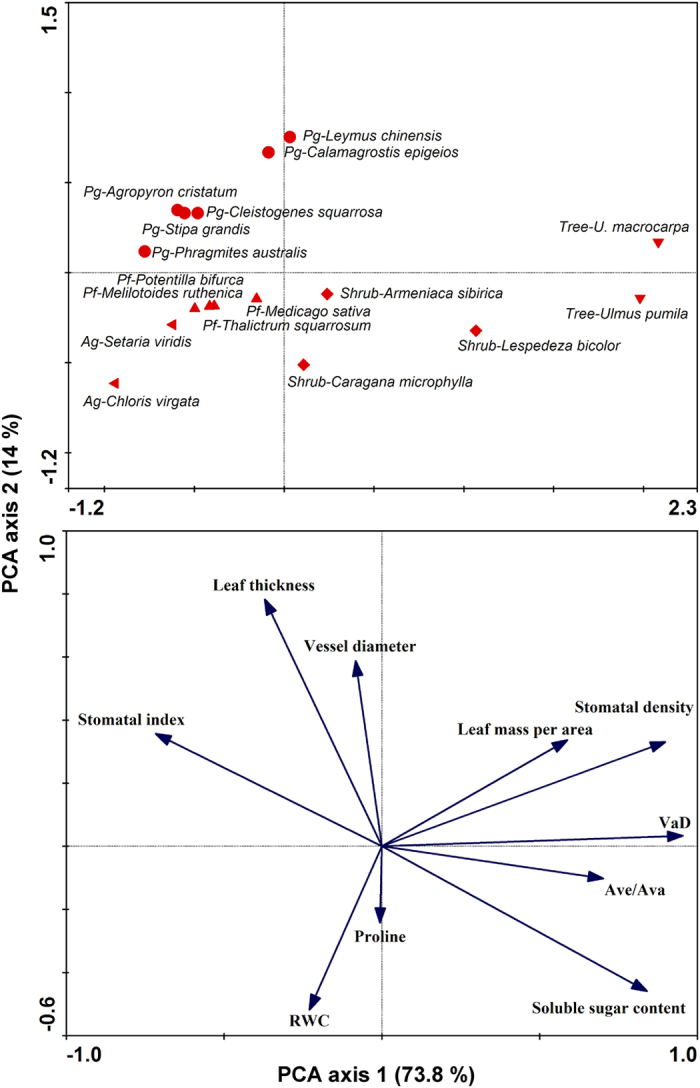
Principal component analysis (PCA) plots showing the separation of leaf morphological, physiological and anatomical traits of measured species for each plant functional type in temperate grasslands along a large-scale aridity gradient in northeast of China. RWC, leaf relative water content; A_Ve_/A_Va_, ratio of leaf vessel area to vascular area; Pg, perennial grasses; Ag, annual grasses; Pf, perennial forbs.

Along the large-scale gradient (from site 1 to 9), the mean annual standardized precipitation-evapotranspiration index (SPEI) gradually decreased, reflecting an increase in aridity (Table 1; Fig. 1). In a principal components analysis (PCA) of the 10 leaf traits in 17 species, the first and second axes accounted for 87.8% of the total variation across the aridity gradient. The first axis, which explained 73.8% of the variation in leaf traits, was primarily related to LMA, stomatal density, vascular diameter, leaf soluble sugar content and stomatal index. The second axis, which explained 14% of the variation in leaf traits, was mainly associated with leaf thickness, vessel diameter, leaf relative water content (RWC) and proline content. In general, functional types grouped separately along the first and second axes, and leaf morphological, physiological and anatomical traits of individual selected species were similar to those of other species in the same PFT (*i*.*e*., trees, shrubs, perennial grasses, annual grasses and perennial forbs) (Fig. 2).

### Morphological traits

**Figure 3:**
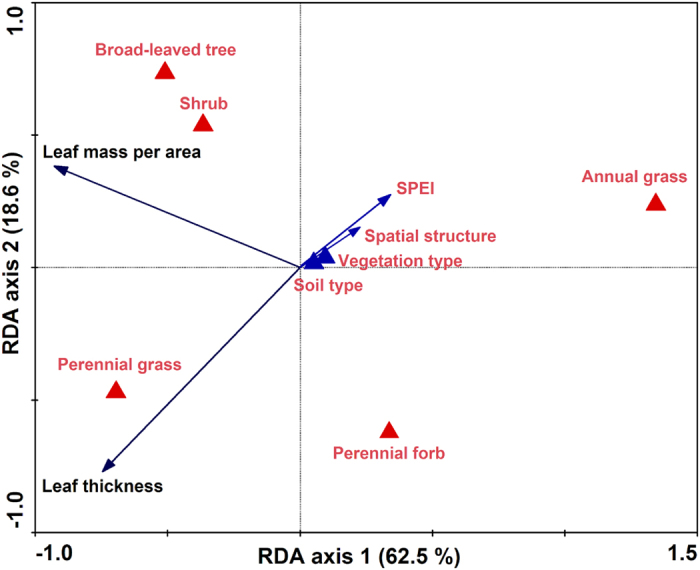
Redundancy analysis (RDA) biplot showing the effects of standardized precipitation-evapotranspiration index (SPEI), spatial structure, soil type, vegetation type and plant functional types on leaf thickness and leaf mass per area along the large-scale aridity gradient, northeastern China. Soil type, vegetation type and plant functional types (qualitative factors) are indicated by triangles; SPEI and spatial structure (quantitative factors), leaf thickness and leaf mass per area are indicated by arrows.

The first axis of RDA ordination, which explained 62.5% of the variation in thickness and LMA, was mainly associated with SPEI. The second axis, which described 18.6% of the variation in thickness and LMA, was primarily related to PFTs. Spatial structure, soil type and vegetation type did not show strong relationships with leaf morphological traits. Among the five PFTs, leaf thickness in perennial grasses was significantly higher than for the other PFTs (Fig. 3). Leaf thickness in shrubs (R = −0.501, *P* < 0.01), perennial grasses (R = −0.307, *P* < 0.05) and forbs (R = −0.298, *P* < 0.05) increased gradually with decreasing SPEI (or increasing aridity) along the large-scale gradient (Tables 2 and 3). Leaf thickness in shrubs at site 7 (moderate aridity) was 158.1% (*F* = 121.75, *P* < 0.001) higher than that at site 1 (no aridity) (File S1).

As shown in the RDA ordination, the LMA in trees, shrubs and perennial grasses were remarkably greater than that in annual grasses and perennial forbs (Fig. 3; File S1). Partial correlation analysis and the mixed effects model revealed that LMA in perennial grasses increased (R = −0.498, *P* < 0.01), while that in trees decreased with decreasing SPEI along the gradient (R = 0.515, *P* < 0.01; Tables 2 and 3).

### Physiological adjustment

**Figure 4:**
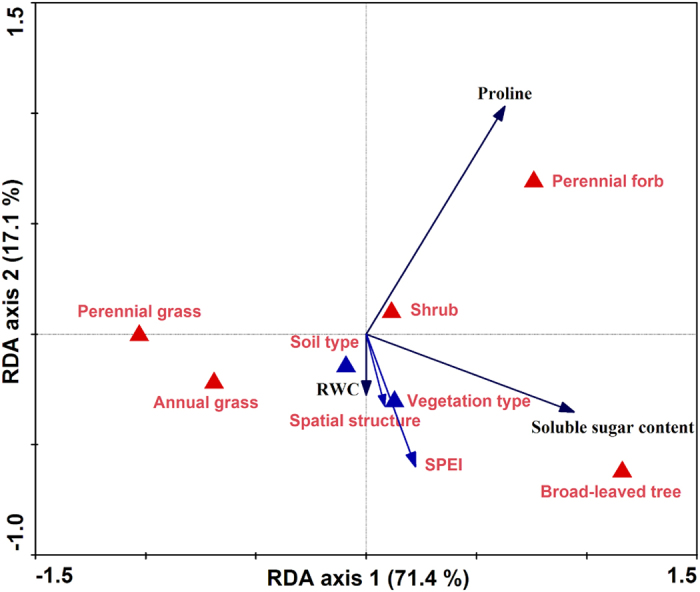
Ordination biplot of redundancy analysis (RDA) displaying the effects of standardized precipitation-evapotranspiration index (SPEI), spatial structure, soil type, vegetation type and plant functional types on leaf relative water content (RWC), proline and soluble sugar contents along the large-scale aridity gradient, northeastern China. Soil type, vegetation type and plant functional types (qualitative factors) are indicated by triangles; SPEI and spatial structure (quantitative factors), RWC, proline and soluble sugar contents are indicated by arrows.

The first axis of RDA ordination, which explained 71.4% of the variation in leaf RWC, proline and soluble sugar contents, was mainly related to PFTs. The second axis, which described 17.1% of the variation in leaf RWC, proline and soluble sugar contents, was primarily associated with SPEI among sites. Spatial structure, soil type and vegetation type did not show strong relationships with leaf physiological traits. Leaf RWC in trees, perennial and annual grasses were remarkably greater than that in shrubs and perennial forbs (Fig. 4). Leaf RWC in each PFT fluctuated significantly and was not correlated with SPEI across the aridity gradient (File S2; Table 2).

The RDA ordination revealed that shrubs and perennial forbs had higher proline contents than the other three PFTs, and that trees had a higher soluble sugar content than that of the other four PFTs (Fig. 4). Partial correlation analysis and the mixed effects model revealed that proline content in trees declined (R = 0.342, *P* < 0.05), while soluble sugar content increased with increasing aridity along the gradient (R = −0.330, *P* < 0.05; Tables 2 and 3).

Unlike trees, shrubs exhibited opposite patterns in proline and soluble sugar contents along the gradient (Fig. 4; Tables 2 and 3). Specifically, the proline content in shrubs at site 7 (moderate aridity) was 188.3% higher than that at site 1 (no aridity) (*F* = 177.25, *P* < 0.001). In addition, the proline content in perennial and annual grasses increased with increasing aridity, while that in perennial forbs was not correlated with SPEI among sites (File S2; Tables 2 and 3).

### Anatomical structures

**Figure 5:**
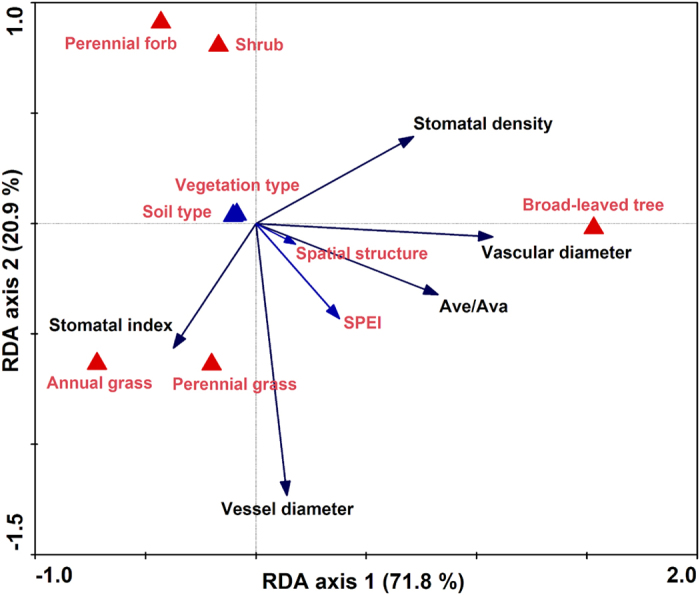
Redundancy analysis (RDA) biplot showing stomatal density, stomatal index, vessel diameter, vascular diameter and ratio of vessel area to vascular area (A_Ve_/A_Va_) responses to standardized precipitation-evapotranspiration index (SPEI), spatial structure, soil type, vegetation type and plant functional types along the large-scale aridity gradient, northeastern China. Soil type, vegetation type and plant functional types (qualitative factors) are indicated by triangles; SPEI and spatial structure (quantitative factors), stomatal density, stomatal index, vessel diameter, vascular diameter and A_Ve_/A_Va_ are indicated by arrows.

The first and the second axes of RDA ordination explained 71.8% and 20.9% of the variation in leaf stomatal density and index, vascular and vessel diameters, and A_Ve_/A_Va_ across the large-scale aridity gradient. Spatial structure, soil type and vegetation type did not show strong relationships with leaf anatomical traits. In the five PFTs, trees had higher average stomatal density than that in shrubs, perennial grasses, annual grasses and perennial forbs (Fig. 5, File S3). However, stomatal density in each PFT fluctuated significantly and was not correlated with SPEI along the gradient (File S3). Unlike stomatal density, the stomatal index in shrubs (R = 0.425, *P* < 0.001) and perennial grasses (R = 0.307, *P* = 0.022) decreased with increasing aridity (Table 2).

RDA ordination revealed that leaf vascular diameter in trees was considerably greater than that in the other four PFTs among sites. In contrast, vessel diameter of the perennial and annual grasses was greater than that in shrubs, perennial forbs and trees (Fig. 5). Partial correlation analysis and mixed effects model indicated that vessel diameter of shrubs (R = 0.373, *P* < 0.001) and perennial grasses (R = 0.374, *P* < 0.001) were strongly and positively related to SPEI (Tables 2 and 3). In addition, trees maintained relatively higher A_Ve_/A_Va_ ratios than other PFTs along the gradient (File S4). The ratio of A_Ve_/A_Va_ in perennial grasses (R = 0.344, *P* < 0.01) and forbs (R = 0.308, *P* < 0.05) gradually decreased with increasing aridity (Table 2).

## Discussion

Water deficit is the major abiotic factor affecting plant survival, distribution, growth and reproduction in the northeast of China^9^,^26^. Exploring the critical factors regulating trait variations of PFTs and testing the strategy divergences of PFTs along large-scale aridity gradients are important challenges in plant ecology. In this study, PCA analyses showed that the aridity effects on single selected species are similar to those of other species in the same PFT along the aridity gradient in temperate grasslands. Therefore, selecting PFT to represent groups of plant species can reduce the complexity associated with studying major ecosystem processes. Accordingly, this knowledge is essential to our ability to predict the fate of natural ecosystems during long-term climate changes.

One way plants respond to long-term climate changes is through environmentally induced shifts in phenotype (phenotypic plasticity)^27^. In the present study, leaf thickness in shrubs, perennial grasses and forbs increased gradually with increasing aridity along the gradient (File S1). Greater leaf thickness can provide a structural basis for water storage and is evolutionarily favored for efficient water use. These results are consistent with previous observations showing that high leaf thickness enables plants to maintain a relatively high leaf water content during water scarcity^28^,^29^,^30^,^31^. In addition, we found that leaf thickness in annual grasses was the smallest among the five PFTs and showed no significant differences along the gradient. This might have been due to annual grasses mainly growing during rainy seasons and using seasonal precipitation efficiently in dry regions. Similarly, previous studies showed that the leaves of annual species were slightly thinner than those of perennial species^32^,^33^.

Relatively high LMA in perennial grasses at the dry sites (sites 5–9) might indicate their high tolerance to increasing aridity (File S1). These results were similar to those reported in temperate grasslands^18^,^29^, shrublands in the Mediterranean^34^ and tropical dry forests^35^ along large-scale aridity gradients. Higher LMA has been shown to be the main adaptive strategy to water scarcity, which is positively related to allocation of greater leaf structural strength and photosynthetic tissue per area, contributing to higher tolerance to drought conditions^36^,^37^. However, higher LMA did not enhance the capacity to maintain high leaf RWC in perennial grasses, because LMA was not significantly related to RWC across the gradient (Table 2). These findings suggest that perennial grasses might allocate investment in structural tissues rather than physiological regulatory substances to cope with water scarcity in arid regions.

Plants can endure aridity stress by adjusting physiological traits to maintain tissue water potential as high as possible. There has been a great deal of research showing that free proline and soluble sugar accumulation in plants are the crucial osmotic adjustment processes to adapt to aridity, including both short-term and long-term stresses^38^,^39^. Proline and soluble sugar accumulation enables cells to maintain turgor, thus allowing high water content levels to be sustained whilst cell osmotic potential decreases under water deficient conditions^1^,^14^,^40^,^41^. In this study, although LMA and proline content in trees decreased with increasing aridity, their soluble sugar content increased simultaneously (Figs 3 and 4; File S1 and 2). The high soluble sugar content in trees during aridity stress enhanced their capacity to maintain relatively high leaf RWC (Table 2). However, the opposite patterns in proline and soluble sugar contents were observed in shrubs along the gradient (Fig. 2). These findings indicate that the adjustment functions of proline and soluble sugar are different in trees and shrubs. Trees, shrubs and perennial forbs had relatively higher proline and soluble sugar contents than the two grass PFTs (Fig. 4). These results suggest that their capacity for osmotic adjustment was greater than that of perennial and annual grasses in the regions.

Changes in anatomic structure reflect plant evolutionary adaptation to long-term environmental stresses. Plant stomata not only prevent desiccation, but also dynamically regulate water loss to maintain efficient daytime water use^42^. In the present study, stomatal densities of the five PFTs were not significantly correlated with water availability along the gradient; however, stomatal index in shrubs and perennial grasses decreased considerably with increasing aridity (Table 2). These findings are consistent with those of previous studies in which relative lower stomatal indexes were found to enable plants to minimize water loss through leaf transpiration and to increase water use efficiency^43^,^44^. The significantly lower stomatal index in shrubs and perennial grasses under drought stress (File S3) underlie the two PFTs maintaining high capacity to regulate water loss by leaf transpiration.

At the anatomical level, tolerance to water scarcity depends on a variety of factors, including leaf vessel diameter, vessel length, A_Ve_/A_Va_, resistance of end wall plates and pathway redundancy^45^. The highest average leaf A_Ve_/A_Va_ among the PFTs was for trees. This indicated that the large and dense xylem vessels allowed trees to have a strong water transport capacity and low investment of energy along the gradient. Whether there is a negative relationship between vessel diameter and water flow is still the subject of debate, mainly because of variations in evidence from different plant species or PFTs. In our observations, vessel diameter in shrubs and perennial grasses decreased with increasing aridity, while that in trees and annual grasses exhibited slight variations along the gradient (File S4). Under water scarce conditions, shrubs and perennial grasses develop narrow vessels that maximize water uptake and favor plant survival^8^. The relatively smaller variations in vessel diameter in annual grasses and trees were likely because native annual grasses combine short life cycles and high rates of leaf gas exchange when utilizing maximum water resources efficiently during rainy seasions^1^, and because deep-rooted trees (*i*.*e*., the length of root > 3 m) could utilize soil moisture of the deep soil layer^46^,^47^. Therefore, these findings indicate that aridity may not be the primary driver governing anatomical structure of trees and annual grasses in the temperate grasslands along the large-scale gradient in northeastern China.

In conclusion, significant relationships between PFT traits (*e*.*g*., leaf thickness in perennial grasses and forbs, vessel diameter in shrubs and perennial grasses, proline content in perennial and annual grasses) and SPEI were observed along the aridity gradient. Shrubs and perennial grasses showed the greatest responses to variability of aridity through regulation of leaf morphological, physiological and anatomical traits, followed by perennial forbs. Trees and annual grasses endured water scarcity through only physiological regulation. These findings support our hypothesis that water availability is a critical factor driving trait variations of PFTs and that there are significant divergences in leaf morphological, anatomical and physiological traits among PFTs in temperate grasslands along the large-scale aridity gradient. Divergences in adaptive strategies under aridity among these PFTs were likely caused by their differences in utilization of water resources with different temporal and spatial distribution patterns.

## Materials and Methods

### Study sites

Nine sites were selected for plant sampling which occurred from June to August in 2010 and 2011 along a large-scale longitudinal climate gradient (44°34′ to 43°36′N; 114°34′ to 124°19′E, about 900 km long) across Jilin province and Inner Mongolia in northeast China (Table 1; Fig. 1). The typical climate in this area is continental monsoon, with large variations in seasonal temperature and precipitation. Mean annual temperature and precipitation along the gradient range from approximately 1.2 °C to 6.3 °C and 240 to 476 mm, respectively (from a long-term database, 1981–2010; http://www.cma.gov.cn/). The precipitation gradient dominates vegetation zonality and plant distributions in this region^9^,^26^. Due to the sharp decline in precipitation from east to west, vegetation varies gradually from moist meadows (site 1–4) in the east to typical steppes (site 5–7) and desert steppes (site 8–9) in the west, with agricultural fields, shrubs and woods in the middle. Soils in most sites are dark meadow soil and chernozem in the east and chernozem and chestnut in the west. The sites had not been grazed, ploughed, fertilized or burned for at least 10 years prior to 2011, but transient floods may have occurred in the eastern meadows^26^. Detailed information regarding the measured species, total species number and percentage of measured species for each PFT at 9 sites are listed in the supporting information (File S5).

### Experimental design

Based on plant size, life form, life history, bio-climatic tolerance, as well as their responses to the large-scale aridity gradient^48^, plant species were grouped into five main PFTs: temperate cold-deciduous broad-leaved trees (*Ulmus pumila* L. and *U. macrocarpa* Hance.), temperate cold-deciduous low or high shrubs (*Armeniaca sibirica* (L.) Lam., *Caragana microphylla* Lam. and *Lespedeza bicolor* Turcz.), perennial grasses (*Leymus chinensis* (Trin.) Tzvel., *Agropyron cristatum* (L.) Gaertn., *Stipa grandis* P. Smirn., *Calamagrostis epigeios* (L.) Roth., *Cleistogenes squarrosa* (Trin.) Keng. and *Phragmites australis* (Cav.) Trin.), annual grasses (*Setaria viridis* (L.) Beauv. and *Chloris virgata* Swartz.) and perennial forbs (*Thalictrum squarrosum* Steph., *Potentilla bifurca* L., *Melilotoides ruthenica* (L.) Sojak. and *Medicago sativa* L.). These species are representative of the region’s PFTs^15^, and the five PFTs are the typical plant function types in local vegetation.

A typical native grassland of about 1–2 ha was selected for investigation of perennial grasses and forbs and annual grasses sampling at each site. Four plots (20 × 20 m each) with uniform vegetation and soil texture were established randomly per site and surveyed at the time of the expected peak of vegetation development in the corresponding location. Plants in each plot were sampled using 4–5 randomly located 1 × 1 m quadrats. Trees and shrubs were sampled at each site in 3–4 plots (1–2 ha each) with uniform soil texture that were established randomly using 4–5 randomly located 10 × 10 m or 4 × 4 m quadrats. The sampling plot and quadrat sizes were primarily selected based on plant sizes. From within each plot, 8–16 soil core samples were collected randomly and used for determination of soil properties.

### Leaf relative water content and mass per area measurements

Within each site, the second fully expanded leaf (hereafter referred to as the second leaf from the shoot top) of all species was sampled randomly from 3–5 mature plants in each quadrat at about 9:00 a.m., and then weighted immediately in the field to obtain the fresh weight (FW). The leaves were then placed into plastic bags (100 ml) filled with distilled water and allowed to stand overnight. The turgid fresh weight (TW) of each leaf was measured the next morning. Leaf samples were placed in perforated paper bags, oven-dried at 80 °C for 24 h and weighed to measure the dry weight (DW). The relative water content (RWC) was calculated as RWC = (FW − DW)/(TW − DW)^49^. LMA was measured using a flatbed scanner connected to a personal computer running image analysis software. LMA denotes leaf dry mass per area^50^.

### Leaf anatomy

A total of 5–6 sections (1 × 1 cm) were cut from the middle of mature leaves in each quadrat (one section per plant) and fixed in FAA (3.7% formalin, 50% ethanol and 5% acetic acid) for each species respectively. Leaf cross sections of 8–10 μm were obtained with a rotary microtome (Leica, RM2235, Germany), after which leaf thickness, diameter of vessel and vascular bundles, as well as the vessel and vascular area were measured by the NIS-Elements Documentation (Nikon, Japan). A_Ve_/A_Va_ was calculated by vessel area/vascular area.

Leaf pieces (1 × 1 cm) were treated with NaClO to eliminate the mesophyllic tissue. Once the two epidermises were separated and the remaining leaf mesophyll was removed, the pieces were stained with safranin and mounted in glycerol. After these leaf preparations were made, the epidermal cell and abaxial stomata were counted for the leaf samples^17^. The stomatal index refers to the ratio of stomata number to the total number of epidermal cells.

### Proline and soluble sugar contents

Five samples (10 g per sample, collected from five plants, respectively) were taken in each quadrat for each species, respectively. The samples were oven-dried at 80 °C for 24 h to constant weight and then ground to pass through a 100 mesh screen. Next, 0.5 g powdered samples were added to sulphosalicylic acid (10 ml, 3%), and the extract was filtered through filter paper. Finally, 2 ml aliquots were taken for proline estimation by the acid-ninhydrin method^51^.

Leaf powders of each sample (50 mg) were extracted with 80% ethanol (v/v) at 85 °C for 1 h. The solutions were then centrifuged at 12,000 g for 10 min. Ethanol extractions were repeated three times, and the three supernatants from extraction were combined. The supernatants were then treated with activated charcoal and evaporated to dryness in a vacuum evaporator. Finally, residues were dissolved in distilled water and subjected to soluble sugar analysis using the anthrone-sulfuric acid method^52^.

### Aridity data

The standardized precipitation-evapotranspiration index (SPEI) has been proposed to quantify the aridity condition over a given area. The SPEI considers not only precipitation, but also evapotranspiration (PET) data, allowing for a more complete approach to explore the effects of climate change on aridity conditions. The SPEI was calculated by the SPEI-package for R (R Development Core Team, USA) on the basis of monthly precipitation, monthly-mean temperature, and monthly mean sunshine hours data recorded at 9 sites along the gradient from 1981 to 2010 with the scales of 3-month (June, July and August) and 12-month. Long-term (1981–2010) climate data were obtained from the China Meteorological Administration (http://www.cma.gov.cn/) and Worldclim-Global Climate Data (http://www.worldclim.org/). The 3-month SPEI reflected aridity condition during the summer (from June to August), and the 12-month SPEI reflected long-term water balance patterns. The aridity grade in this region was classified according to the Chinese classification of meteorological aridity (no aridity, −0.5 < SPEI; light aridity, −1 < SPEI ≤ −0.5; moderate aridity, −1.5 < SPEI ≤ −1; severe aridity, −2 < SPEI ≤ −1.5; and extreme aridity SPEI ≤ −2). Accordingly, sites 1–2 are classified as no aridity, sites 3–5 as light aridity, sites 6–7 as moderate aridity and sites 8–9 as severe aridity (Table 1).

### Statistical analysis

A principal component analysis (PCA), implemented through the Canoco for Windows 4.5 package (Ithaca, NY, USA) was used to summarize leaf morphological, physiological and anatomical traits of the single selected plant species and determine if it was similar to its PFT. Constrained ordination model–redundancy analyses (RDA) were conducted using the Canoco for Windows 4.5 package, with SPEI, PFTs, spatial structure, soil type and vegetation type as explanatory variables, and leaf morphological, physiological and anatomical traits of the five PFTs as response variables. The response variables were log-transformed (X′ = log_10_10 * X + 1), centered and standardized to zero mean. Qualitative factors were coded for the program using a set of ‘dummy factors’^53^. Spatial structure (x, y, xy, x^2^, y^2^, x^2^y, xy^2^, x^3^, y^3^) consisted of 9 terms, in which latitudinal (x) and longitudinal (y) coordinates were used to calculate a cubic trend surface^54^.

Partial correlation was used to quantify relationships between leaf traits and SPEI while controlling the effects of additional variables (soil type and spatial structure). Differences in each parameter among sites were tested by one-way analysis of variance (ANOVA). The explanatory power of SPEI on leaf morphological, physiological and anatomical traits in PFTs was tested using linear mixed-effects models and incorporating soil type and spatial structure as random effects by SPSS 21 (SPSS for Windows, Chicago, IL, USA).

## Additional Information

**How to cite this article**: Guo, C. *et al*. Morphological, physiological and anatomical traits of plant functional types in temperate grasslands along a large-scale aridity gradient in northeastern China. *Sci. Rep.* **7**, 40900; doi: 10.1038/srep40900 (2017).

**Publisher's note:** Springer Nature remains neutral with regard to jurisdictional claims in published maps and institutional affiliations.

## Supplementary Material

Supporting Information

## Figures and Tables

**Table 1:** Information regarding the study sites (1–9) including data on location, vegetation type, climatic index, soil type and plant functional types (PFTs) in temperate grasslands along a large-scale aridity gradient in northeast China.

Sites	No.	Location	ELE (m)	Vegetation type	MAP (mm)	MAT (°C)	SPEI-3	SPEI	Soil type	PFTs
Wulantuga	1	44°35′N; 124°19′E	209	Meadow	476	5.8	1.13	−0.26 (NA)	Dark meadow	Tr, Sh, Pg, Ag, Pf
Yaojingzi	2	44°36′N; 123°31′E	139	Meadow	440	5.6	1.05	−0.41 (NA)	Dark meadow	Tr, Sh,Pg, Ag, Pf
Huatugula	3	43°54′N; 122°04′E	191	Meadow	399	6.0	0.98	−0.62 (LA)	Dark meadow	Tr, Sh, Pg, Ag, Pf
Shaogen	4	43°38′N; 120°49′E	304	Meadow	383	6.3	0.9	−0.74 (LA)	Chernozem	Tr, Sh,Pg, Ag, Pf
Lindong	5	43°53′N; 119°22′E	625	Steppe	375	5.2	0.83	−0.85 (LA)	Chernozem	Tr, Sh, Pg, Ag, Pf
Linxi	6	43°34′N; 118°00′E	924	Steppe	374	4.8	0.7	−1.03 (MA)	Chernozem	Tr, Sh, Pg, Ag, Pf
Aqiwula	7	43°38′N; 116°42′E	1219	Steppe	320	2.7	0.61	−1.32 (MA)	Chestnut	Tr, Sh, Pg, Ag, Pf
Dabuxiletu	8	43°56′N; 115°46′E	1143	Desert steppe	278	2.1	0.58	−1.54 (SA)	Chestnut	Sh, Pg, Ag
Baogedawula	9	43°56′N; 114°34′E	1092	Desert steppe	240	1.2	0.55	−1.68 (SA)	Chestnut	Sh, Pg, Ag

ELE, elevation; MAP, mean annual precipitation; MAT, mean annual temperature; SPEI-3, mean standardized precipitation-evapotranspiration index in June, July and August; SPEI, mean annual standardized precipitation-evapotranspiration index; NA, no aridity; LA, light aridity; MA, moderate aridity; SA, severe aridity; Tr, temperate cold-deciduous broad-leaved tree; Sh, temperate cold-deciduous low or high shrub; Pg, perennial grass; Ag, annual grass; Pf, perennial forb.

**Table 2:** Partial correlations between standardized precipitation-evapotranspiration index (SPEI) and leaf morphological, physiological and anatomical traits in five plant functional types along the large-scale aridity gradient in northeast China while controlling the effects of spatial structure and soil type.

		Tr	Sh	Pg	Ag	Pf
LMA	SPEI	**0**.515**	−0.241	**−0**.498**	−0.291	−0.127
LT	SPEI	−0.278	**−0**.501**	**−0**.307*	0.076	**−0**.298*
VaD	SPEI	−0.284	0.286	0.094	−0.015	−0.094
VeD	SPEI	−0.026	**0**.373**	**0**.374**	0.047	**0**.316*
A_Ve_/A_Va_	SPEI	−0.291	0.224	**0**.344**	0.202	**0**.308*
SD	SPEI	0.289	0.116	0.083	−0.017	0.208
SI	SPEI	0.253	**0**.425**	**0**.307*	−0.032	0.172
RWC	SPEI	−0.274	0.131	0.275	0.281	0.164
Pr	SPEI	**0**.344*	**−0**.517**	**−0**.521**	**−0**.587**	0.101
SS	SPEI	**−0**.330*	**0**.504**	−0.031	−0.094	0.073

Dependent variables: LMA, leaf mass per area; LT, leaf thickness; VaD, vascular diameter; VeD, vessel diameter; A_Ve_/A_Va_, ratio of vessel area to vascular area; SD, stomatal density; SI, stomatal index; RWC, leaf relative water content; Pr, proline content; SS, soluble sugar content. Tr, temperate cold-deciduous broad-leaved tree; Sh, temperate cold-deciduous low or high shrub; Pg, perennial grass; Ag, annual grass; Pf, perennial forb. Negative values refer to negative relationships between the examined dependent variables and SPEI. *P < 0.05, **P < 0.01.

**Table 3:** Results (degrees of freedom, *F*- and *P*-values) of linear mixed effects model among standardized precipitation-evapotranspiration index (SPEI) and leaf morphological, physiological and anatomical traits in five plant functional types (PFTs) along the large-scale aridity gradient in northeast of China.

PFTs	LMA	LT	VaD	VeD	A_Ve_/A_Va_	SD	SI	RWC	Pr	SS
**Trees**										
*df*	6	6	6	6	6	6	6	6	6	6
*F*	**30**.**23**	2.79	3.19	4.17	3.93	4.53	1.52	**5**.**78**	**4**.**52**	**17**.**35**
*P*	<**0.001**	0.197	0.081	0.069	0.111	0.051	0.213	**0**.**031**	**0**.**048**	**<0**.**001**
**Shrubs**										
*df*	8	8	8	8	8	8	8	8	**8**	**8**
*F*	**14**.**57**	**29**.**49**	**21**.**67**	**23**.**97**	**141**.**28**	**8**.**19**	**11**.**60**	1.59	**62**.**40**	**58**.**97**
*P*	**<0**.**001**	**<0**.**001**	**<0**.**001**	**<0**.**001**	**<0**.**001**	**<0**.**001**	**<0**.**001**	0.177	**<0**.**001**	**<0**.**001**
**Perennial grasses**										
*df*	8	8	8	8	8	8	8	8	8	8
*F*	**42**.**69**	**7**.**39**	1.94	**10**.**65**	**35**.**88**	**43**.**46**	**10**.**29**	**5**.**55**	**53**.**76**	1.24
*P*	**<0**.**001**	**<0**.**001**	0.065	**<0**.**001**	**<0**.**001**	**<0**.**001**	**<0**.**001**	**0**.**002**	**<0**.**001**	0.291
**Annual grasses**										
*df*	8	8	8	8	8	8	8	8	8	8
*F*	4.34	0.197	4.18	3.29	3.08	3.67	1.24	**16**.**09**	**45**.**72**	0.43
*P*	0.060	0.977	0.071	0.125	0.133	0.079	0.231	**<0**.**001**	**<0**.**001**	0.857
**Perennial forbs**										
*df*	6	6	6	6	6	6	6	6	6	6
*F*	3.62	2.01	**9**.**18**	**18**.**20**	**24**.**96**	4.03	1.19	1.87	2.36	**5**.**15**
*P*	0.074	0.080	**<0**.**001**	**<0**.**001**	**<0**.**001**	0.088	0.329	0.119	0.052	**0**.**047**

Fixed factor: SPEI; random factors: spatial structure, soil type. Dependent variables: LMA, leaf mass per area; LT, leaf thickness; VaD, vascular diameter; VeD, vessel diameter; A_Ve_/A_Va_, ratio of vessel area to vascular area; SD, stomatal density; SI, stomatal index; RWC, relative water content; Pr, proline content; SS, soluble sugar content.
