# Supplementary material for: Morphological, physiological and anatomical traits of plant functional types in temperate grasslands along a large-scale aridity gradient in northeastern China
Source: Sci Rep. 2017 Jan 20;7:40900. doi: 10.1038/srep40900 (PMC5247725; doi:10.1038/srep40900)
Supplement: Supporting Information [file srep40900-s1.doc]

**Morphological, physiological and anatomical traits of plant functional types in temperate grasslands along a large-scale aridity gradient in northeastern China**

**Chengyuan Guo^1^, Linna Ma^1^, Shan Yuan^1^, Renzhong Wang^1^**

^1^*State Key Laboratory of Vegetation and Environmental Change, Institute of Botany, the Chinese Academy of Sciences,* *20 Nanxincun, Xiangshan, Beijing, 100093, China*

*C. G. and L. M. contributed equally to this work; Correspondence and requests for materials should be addressed to L.M. (Email:* [*maln@ibcas.ac.cn*](mailto:maln@ibcas.ac.cn) Tel: +86-10-62836564 Fax: +86-10-82595962*) or R.W. (Email:* [*wangrz@ibcas.ac.cn*](mailto:wangrz@ibcas.ac.cn) Tel: +86-10-62836550 Fax: +86-10-82595962*).*

**Supporting Information**

**File S1. Leaf thickness (LT) and leaf mass per area (LMA) in plant functional types in the temperate grasslands along a large-scale aridity gradient, northeastern China.** Vertical bars indicate standard errors of means. Tr, temperate cold-deciduous broad-leaved tree; Sh, temperate cold-deciduous low or high shrub; Pg, perennial grass; Ag, annual grass; Pf, perennial forb. Difference lowercase letters indicate statistically significant differences among sites (site/site) for each property within each PFTs (*P* < 0.05).





**File S2. Leaf relative water content (RWC), proline and soluble sugar contents in plant functional types in the temperate grasslands along a large-scale aridity gradient.** Vertical bars indicate standard errors of means. Tr, temperate cold-deciduous broad-leaved tree; Sh, temperate cold-deciduous low or high shrub; Pg, perennial grass; Ag, annual grass; Pf, perennial forb. Difference lowercase letters indicate statistically significant differences among sites (site/site) for each property within each PFTs (*P* < 0.05).


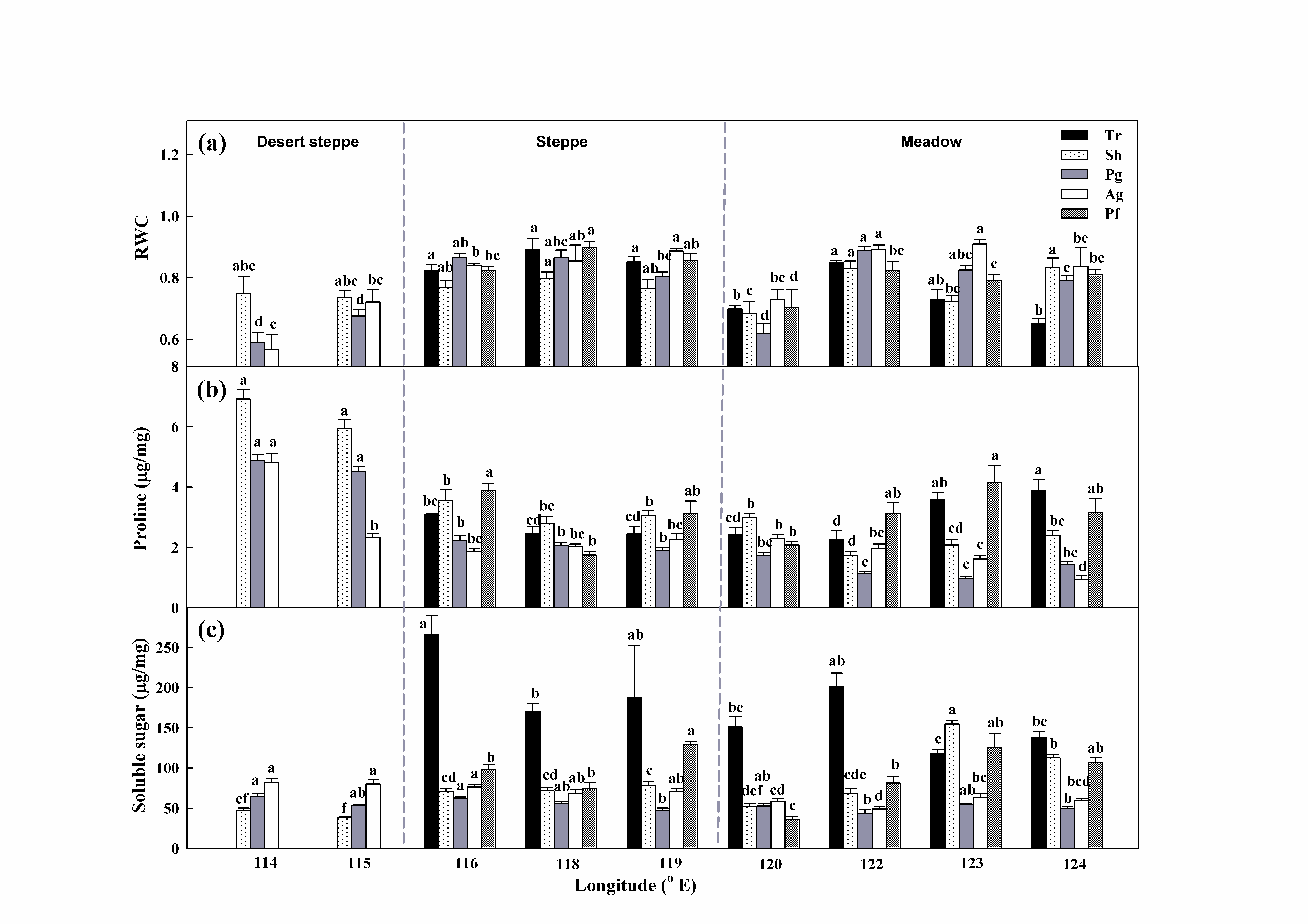


**File S3.** **Stomatal density and stomatal index in 5 plant functional types at 9 sites along the large-scale aridity gradient, northeastern China.** Vertical bars indicate standard errors of means. Tr, temperate cold-deciduous broad-leaved tree; Sh, temperate cold-deciduous low or high shrub; Pg, perennial grass; Ag, annual grass; Pf, perennial forb. Difference lowercase letters indicate statistically significant differences among sites (site/site) for each property within each PFTs (*P* < 0.05).


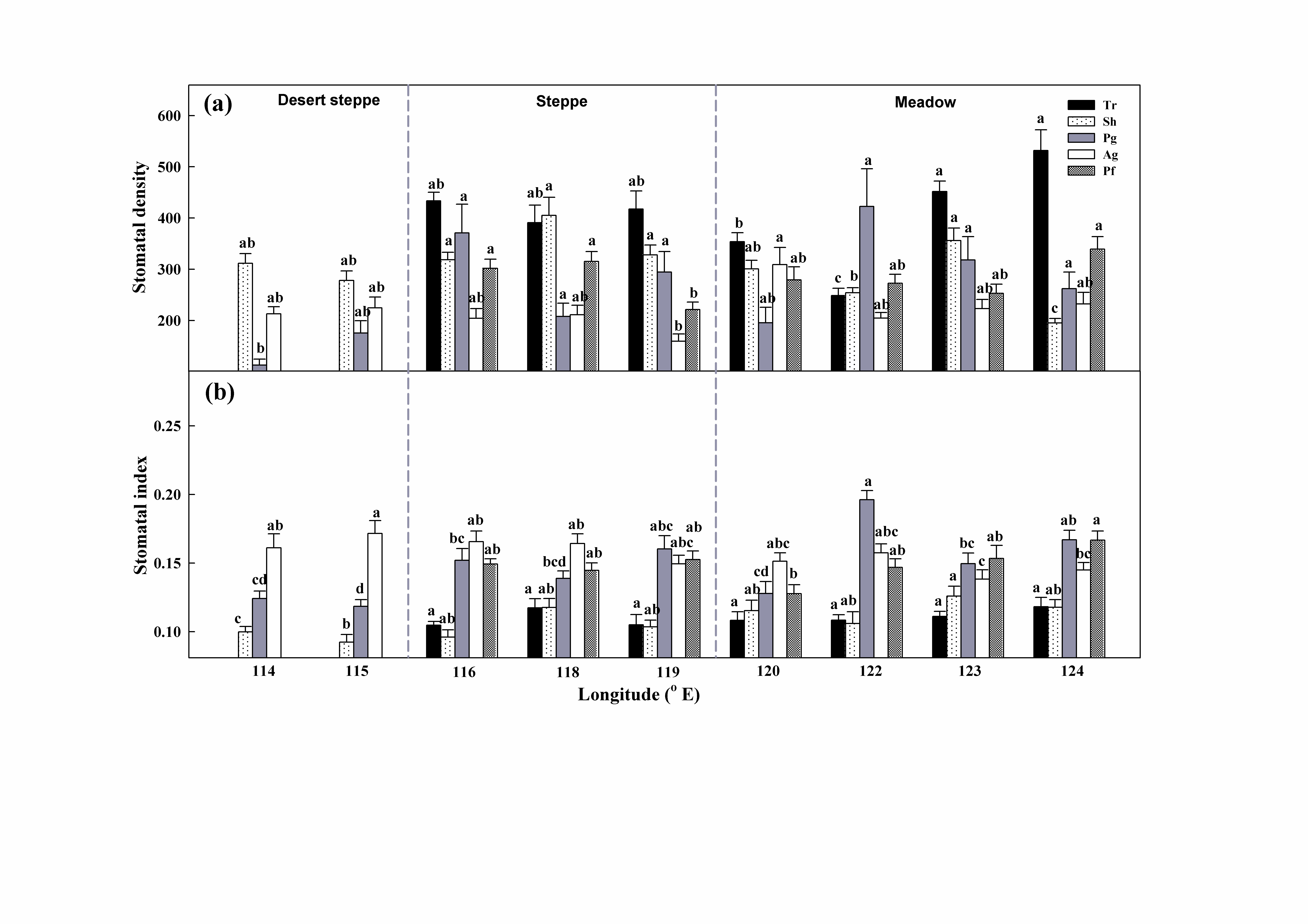


**File S4.** **Vessel diameter (VeD), vascular diameter (VaD) and ratio of vessel area to vascular area (A_Ve_/A_Va_) in plant functional types in temperate grasslands along a large-scale aridity gradient, northeastern China.** Vertical bars indicate standard errors of means. Tr, temperate cold-deciduous broad-leaved tree; Sh, temperate cold-deciduous low or high shrub; Pg, perennial grass; Ag, annual grass; Pf, perennial forb. Difference lowercase letters indicate statistically significant differences among sites (site/site) for each property within each PFTs (*P* < 0.05).


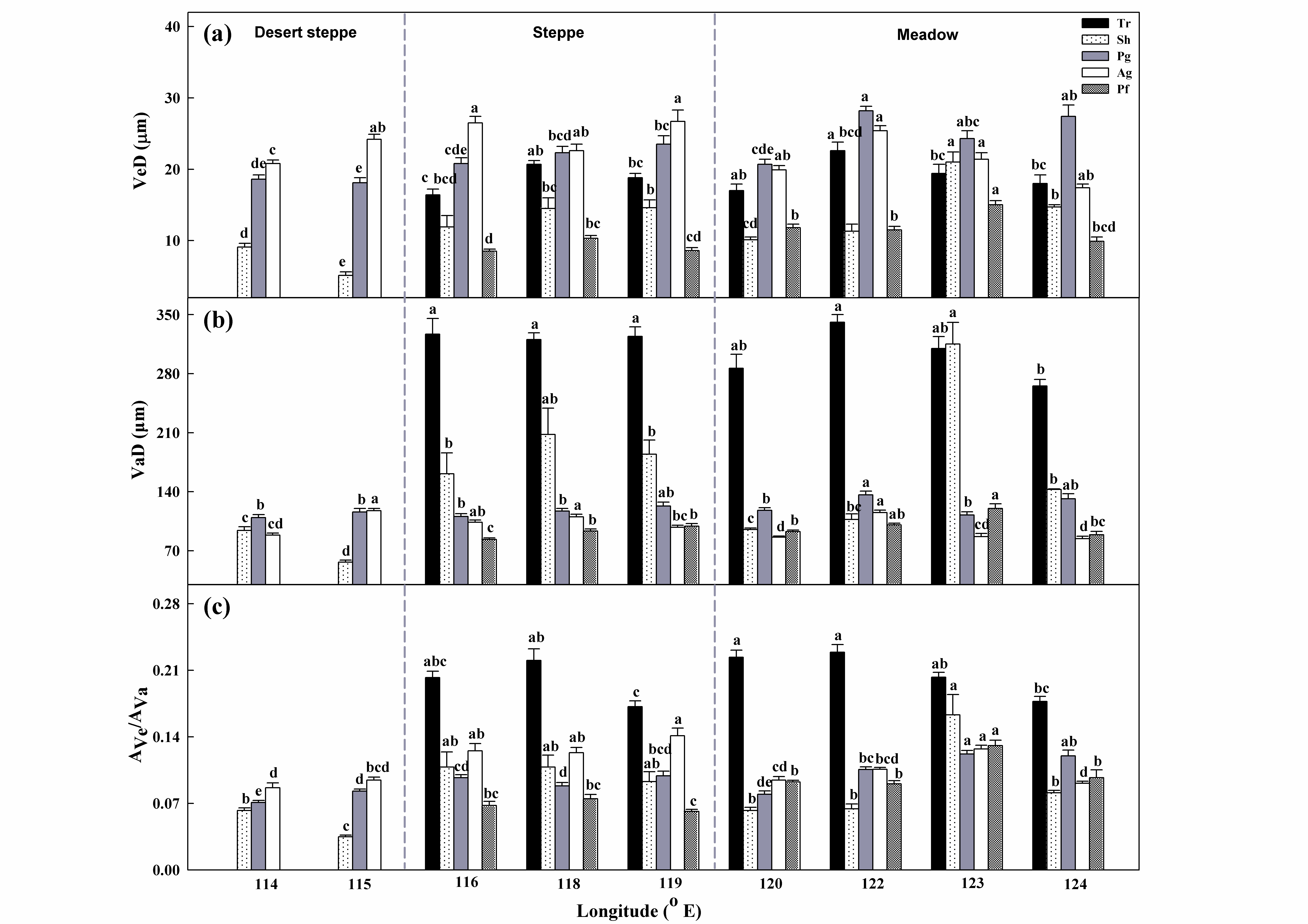


**File S5. Measured species (MS), total species number in community (TSNC) and percentage of measured species (PMS) for each plant functional type (PFT) at 9 sites in temperate grasslands along a large-scale aridity gradient, northeastern China. Tr, temperate cold-deciduous broad-leaved tree; Sh, temperate cold-deciduous low or high shrub; Pg, perennial grass; Ag, annual grass; Pf, perennial forb.**

| Sites | PFTs | MS | TSNC | PMS |
| --- | --- | --- | --- | --- |
| Wulantuga | Tr | *Ulmus pumila* | 2 | 100 % |
|  |  | *U.* *macrocarpa* |  |  |
|  | Sh | *Armeniaca sibirica* | 5 | 60 % |
|  |  | *Caragana microphylla* |  |  |
|  |  | *Lespedeza bicolor* |  |  |
|  | Pg | *Leymus chinensis* | 12 | 50 % |
|  |  | *Agropyron cristatum* |  |  |
|  |  | *Stipa grandis* |  |  |
|  |  | *Calamagrostis epigeios* |  |  |
|  |  | *Cleistogenes squarrosa* |  |  |
|  |  | *Phragmites australis* |  |  |
|  | Ag | *Setaria viridis* | 4 | 50 % |
|  |  | *Chloris virgata* |  |  |
|  | Pf | *Thalictrum squarrosum* | 7 | 57 % |
|  |  | *Potentilla bifurca* |  |  |
|  |  | *Melilotoides ruthenica* |  |  |
|  |  | *Medicago sativa* |  |  |
| Yaojingzi | Tr | *Ulmus pumila* | 4 | 50 % |
|  |  | *U.* *macrocarpa* |  |  |
|  | Sh | *Armeniaca sibirica* | 4 | 75 % |
|  |  | *Caragana microphylla* |  |  |
|  |  | *Lespedeza bicolor* |  |  |
|  | Pg | *Leymus chinensis* | 11 | 55 % |
|  |  | *Agropyron cristatum* |  |  |
|  |  | *Stipa grandis* |  |  |
|  |  | *Calamagrostis epigeios* |  |  |
|  |  | *Cleistogenes squarrosa* |  |  |
|  |  | *Phragmites australis* |  |  |
|  | Ag | *Setaria viridis* | 3 | 67 % |
|  |  | *Chloris virgata* |  |  |
|  | Pf | *Thalictrum squarrosum* | 7 | 57 % |
|  |  | *Potentilla bifurca* |  |  |
|  |  | *Melilotoides ruthenica* |  |  |
|  |  | *Medicago sativa* |  |  |
| Huatugula | Tr | *Ulmus pumila* | 3 | 67 % |
|  |  | *U.* *macrocarpa* |  |  |
|  | Sh | *Armeniaca sibirica* | 7 | 57 % |
|  |  | *Caragana microphylla* |  |  |
|  |  | *Lespedeza bicolor* |  |  |
|  | Pg | *Leymus chinensis* | 11 | 55 % |
|  |  | *Agropyron cristatum* |  |  |
|  |  | *Stipa grandis* |  |  |
|  |  | *Calamagrostis epigeios* |  |  |
|  |  | *Cleistogenes squarrosa* |  |  |
|  |  | *Phragmites australis* |  |  |
|  | Ag | *Setaria viridis* | 2 | 100 % |
|  |  | *Chloris virgata* |  |  |
|  | Pf | *Thalictrum squarrosum* | 8 | 50 % |
|  |  | *Potentilla bifurca* |  |  |
|  |  | *Melilotoides ruthenica* |  |  |
|  |  | *Medicago sativa* |  |  |
| Shaogen | Tr | *Ulmus pumila* | 2 | 100 % |
|  |  | *U.* *macrocarpa* |  |  |
|  | Sh | *Armeniaca sibirica* | 7 | 57 % |
|  |  | *Caragana microphylla* |  |  |
|  |  | *Lespedeza bicolor* |  |  |
|  | Pg | *Leymus chinensis* | 12 | 50 % |
|  |  | *Agropyron cristatum* |  |  |
|  |  | *Stipa grandis* |  |  |
|  |  | *Calamagrostis epigeios* |  |  |
|  |  | *Cleistogenes squarrosa* |  |  |
|  |  | *Phragmites australis* |  |  |
|  | Ag | *Setaria viridis* | 4 | 50 % |
|  |  | *Chloris virgata* |  |  |
|  | Pf | *Thalictrum squarrosum* | 6 | 67 % |
|  |  | *Potentilla bifurca* |  |  |
|  |  | *Melilotoides ruthenica* |  |  |
|  |  | *Medicago sativa* |  |  |
| Lindong | Tr | *Ulmus pumila* | 2 | 100 % |
|  |  | *U.* *macrocarpa* |  |  |
|  | Sh | *Armeniaca sibirica* | 7 | 57 % |
|  |  | *Caragana microphylla* |  |  |
|  |  | *Lespedeza bicolor* |  |  |
|  | Pg | *Leymus chinensis* | 11 | 55 % |
|  |  | *Agropyron cristatum* |  |  |
|  |  | *Stipa grandis* |  |  |
|  |  | *Calamagrostis epigeios* |  |  |
|  |  | *Cleistogenes squarrosa* |  |  |
|  |  | *Phragmites australis* |  |  |
|  | Ag | *Setaria viridis* | 4 | 50 % |
|  |  | *Chloris virgata* |  |  |
|  | Pf | *Thalictrum squarrosum* | 8 | 50 % |
|  |  | *Potentilla bifurca* |  |  |
|  |  | *Melilotoides ruthenica* |  |  |
|  |  | *Medicago sativa* |  |  |
| Linxi | Tr | *Ulmus pumila* | 2 | 100 % |
|  |  | *U.* *macrocarpa* |  |  |
|  | Sh | *Armeniaca sibirica* | 8 | 50 % |
|  |  | *Caragana microphylla* |  |  |
|  |  | *Lespedeza bicolor* |  |  |
|  | Pg | *Leymus chinensis* | 11 | 55 % |
|  |  | *Agropyron cristatum* |  |  |
|  |  | *Stipa grandis* |  |  |
|  |  | *Calamagrostis epigeios* |  |  |
|  |  | *Cleistogenes squarrosa* |  |  |
|  |  | *Phragmites australis* |  |  |
|  | Ag | *Setaria viridis* | 4 | 50 % |
|  |  | *Chloris virgata* |  |  |
|  | Pf | *Thalictrum squarrosum* | 7 | 57 % |
|  |  | *Potentilla bifurca* |  |  |
|  |  | *Melilotoides ruthenica* |  |  |
|  |  | *Medicago sativa* |  |  |
| Aqiwula | Tr | *Ulmus pumila* | 2 | 100 % |
|  |  | *U.* *macrocarpa* |  |  |
|  | Sh | *Armeniaca sibirica* | 7 | 57 % |
|  |  | *Caragana microphylla* |  |  |
|  |  | *Lespedeza bicolor* |  |  |
|  | Pg | *Leymus chinensis* | 11 | 55 % |
|  |  | *Agropyron cristatum* |  |  |
|  |  | *Stipa grandis* |  |  |
|  |  | *Calamagrostis epigeios* |  |  |
|  |  | *Cleistogenes squarrosa* |  |  |
|  |  | *Phragmites australis* |  |  |
|  | Ag | *Setaria viridis* | 4 | 50 % |
|  |  | *Chloris virgata* |  |  |
|  | Pf | *Thalictrum squarrosum* | 5 | 80 % |
|  |  | *Potentilla bifurca* |  |  |
|  |  | *Melilotoides ruthenica* |  |  |
|  |  | *Medicago sativa* |  |  |
| Dabuxiletu | Tr | 0 | 0 | 0 |
|  | Sh | *Caragana microphylla* | 4 | 75 % |
|  |  | *Lespedeza bicolor* |  |  |
|  | Pg | *Leymus chinensis* | 8 | 75 % |
|  |  | *Agropyron cristatum* |  |  |
|  |  | *Stipa grandis* |  |  |
|  |  | *Calamagrostis epigeios* |  |  |
|  |  | *Cleistogenes squarrosa* |  |  |
|  |  | *Phragmites australis* |  |  |
|  | Ag | *Setaria viridis* | 2 | 100 % |
|  |  | *Chloris virgata* |  |  |
|  | Pf | 0 | 0 | 0 |
| Baogedawula | Tr | 0 | 0 | 0 |
|  | Sh | *Caragana microphylla* | 4 | 75 % |
|  |  | *Lespedeza bicolor* |  |  |
|  | Pg | *Leymus chinensis* | 7 | 71 % |
|  |  | *Agropyron cristatum* |  |  |
|  |  | *Stipa grandis* |  |  |
|  |  | *Calamagrostis epigeios* |  |  |
|  |  | *Cleistogenes squarrosa* |  |  |
|  | Ag | *Setaria viridis* | 3 | 67 % |
|  |  | *Chloris virgata* |  |  |
|  | Pf | 0 | 0 | 0 |
